# Low-dose azithromycin prophylaxis in patients with atrial fibrillation and chronic obstructive pulmonary disease

**DOI:** 10.1007/s11739-024-03653-0

**Published:** 2024-05-31

**Authors:** Tommaso Bucci, Dennis Wat, Sarah Sibley, Dan Wootton, David Green, Pasquale Pignatelli, Gregory Y. H. Lip, Freddy Frost

**Affiliations:** 1 Liverpool Centre for Cardiovascular Science at University of Liverpool, Liverpool John Moores University and Liverpool Heart & Chest Hospital, Liverpool, UK; 2 Department of Clinical Internal, Anesthesiological and Cardiovascular Sciences, Sapienza University of Rome, Rome, Italy; 3 Knowsley Community Respiratory Service, Liverpool Heart & Chest Hospital NHS Foundation Trust, Liverpool, UK; 4 Respiratory Department, Liverpool University Hospital NHS Foundation Trust, Liverpool, UK; 5 Institute of Infection, Veterinary and Ecological Sciences, University of Liverpool, Liverpool, UK; 6 Danish Center for Health Services Research, Department of Clinical Medicine, Aalborg University, Aalborg, Denmark

**Keywords:** Atrial fibrillation, Macrolides, Cardiovascular events, COPD, Azithromycin

## Abstract

Low-dose azithromycin prophylaxis is associated with improved outcomes in people suffering frequent exacerbations of chronic obstructive pulmonary disease (COPD), but the use of macrolides in patients with cardiovascular disease has been debated. To investigate the risk of adverse events after COPD exacerbations in patients with atrial fibrillation (AF) treated with azithromycin prophylaxis. Retrospective cohort study within the TriNetX Platform, including AF patients with COPD exacerbations. Risks of primary and secondary outcomes were recorded up to 30 days post-COPD exacerbations and compared between *azithromycin users* and *azithromycin non-users*. The primary outcomes were the risks for a composite of (1) cardiovascular (all-cause death, heart failure, ventricular arrhythmias, ischemic stroke, myocardial infarction, and cardiac arrest), and (2) hemorrhagic events (intracranial hemorrhage (ICH), and gastro-intestinal bleeding). Cox-regression analyses compared outcomes between groups after propensity score matching (PSM). After PSM, *azithromycin users* (*n* = 2434, 71 ± 10 years, 49% females) were associated with a lower 30-day risk of post-exacerbation cardiovascular (HR 0.67, 95% CI 0.61–0.73) and hemorrhagic composite outcome (HR 0.45, 95% CI 0.32–0.64) compared to *azithromycin non-users* (*n* = 2434, 72 ± 11 years, 51% females)*.* The beneficial effect was consistent for each secondary outcomes, except ICH. On sensitivity analyses, the reduced risk of adverse events in *azithromycin users* was irrespective of smoking status, exacerbation severity, and type of oral anticoagulation. Azithromycin prophylaxis is associated with a lower risk of all-cause death, thrombotic and hemorrhagic events in AF patients with COPD. The possible role of azithromycin prophylaxis as part of the integrated care management of AF patients with COPD needs further study.

**Supplementary Information:**

The online version contains supplementary material available at 10.1007/s11739-024-03653-0.

## Introduction

Chronic obstructive pulmonary disease (COPD) in patients with atrial fibrillation (AF) represents one of the most common comorbidities and is responsible for significantly increased risk of cardiovascular events and death [1–4]. Studies that have investigated the risk of cardiovascular events in COPD patients showed that is highest during the first 30 days after acute exacerbations [5, 6]. Low-dose oral azithromycin treatment is recommended by international guidelines in people with COPD who continue to exacerbate despite optimal inhaled therapy due to their proven anti-inflammatory properties and subsequently reduced exacerbation rates [7, 8]. Drugs targeting anti-inflammatory pathways have previously been associated with reduced cardiovascular event rates but concerns regarding the cardiac safety of macrolide antibiotics including azithromycin have previously been raised [9–11]. We, therefore, investigated cardiovascular outcomes of COPD exacerbations in AF patients receiving low-dose azithromycin prophylaxis.

## Methods

### Study design

This was an observational study conducted within TriNetX, a global federated health research network with access to electronic medical records (EMRs) from academic and community hospitals covering approximately 80 million individuals, mainly located in the United States. Within this network, available data include demographics, healthcare utilization data, e.g., emergency department, inpatient and outpatient attendance, diagnoses using International Classification of Diseases, Tenth Revision, Clinical Modification (ICD-10-CM) codes, laboratory results Logical Observation Identifiers Names and Codes, LOINC, and medications. More information can be found in the supplementary material.

### Study population

The searches on the TriNetX online research platform were performed on the 13th of February 2023. Within the TriNetX platform, we included adults diagnosed with COPD exacerbation (ICD-10 code J44.1). Azithromycin users were defined as individuals who had been prescribed azithromycin at least three months prior to the index event and were still prescribed it at the index event. Non-users were those with no history of azithromycin use in the 12 months prior to the index event. Both azithromycin users and non-users were concomitantly treated with inhaled corticosteroids (ICS: fluticasone, budesonide, or beclomethasone) in the year before the index event. The baseline index event was defined as the most recent COPD exacerbation reported in the TriNetX platform.

At the time of the search, 63 participating health care organizations had data available for individuals who met the study inclusion criteria. Characteristics registered before the index event were considered the baseline characteristics. The clinical outcomes were identified via ICD-10-CM codes (Supplementary Table 1). All-cause death was recorded using specific variable code within the TriNetX platform.

### Outcomes

The primary outcome was the 30-day risk of i) a composite outcome of all-cause death, acute heart failure, severe ventricular arrhythmia, ischemic stroke, and myocardial infarction, and ii) a composite of hemorrhagic events of intracranial hemorrhage (ICH) and gastro-intestinal (GI) bleeding. The secondary outcomes of interest were the 30-day risk of each component of the primary outcomes. The 30-day risk of primary and secondary outcomes was further assessed in different time frames: 1–7 days, 8–14 days, and 15–30 days after the acute COPD exacerbation [5, 6, 12]. The 30-day interval was selected to assess the impact of azithromycin prophylaxis on reducing the risk of cardiovascular events associated with COPD exacerbation. Moreover, we performed a number of some sensitivity analyses to assess the robustness of our primary findings.

First, to further investigate the anti-inflammatory efficacy of macrolides we used another “active comparator” by restricting the *azithromycin non-users* to those receiving inhaled corticosteroids (i.e., akin to GOLD E group [7]), and roflumilast, a selective phosphodiesterase-4 inhibitor.

Second. given a previous study reported macrolides may be less effective in reducing the risk of COPD exacerbation in smokers, we performed a further analysis restricted only in patients with labeled as current smokers[13].

Third, we investigated the risk of primary outcome considering only those patients with severe COPD exacerbations requiring emergency department access.

Fourth, we analyzed the risk of adverse events subdividing patients based on the type of oral anticoagulant in *warfarin users* and non-vitamin k antagonist anticoagulant (*NOAC*) *users*.

### Statistical analyses

Baseline characteristics were compared using chi-squared tests for categorical variables and independent-sample t-tests for continuous variables. Absolute standardized mean differences (ASD) were used to show the distribution of demographic and clinical data among the groups and calculated as the difference in the means or proportions of a particular variable divided by the pooled estimate of standardized differences for that variable. Propensity score matching (PSM) 1:1 was used to control the differences in the comparison cohorts. Cohort matching was performed for age at index event, sex, ethnicity, arterial hypertension, diabetes, ischemic heart disease, heart failure, cerebrovascular disease, and cardiovascular medications (β-blockers, antiarrhythmics, diuretics, lipid lowering drugs, antianginals, calcium channel blockers, angiotensin-converting enzyme inhibitors, anticoagulants, and platelet aggregation inhibitors). These variables were chosen because they may influence clinical outcomes. Any baseline characteristic with a Std diff. < 0.100 was considered well matched. After PSM, Cox-regression proportional hazard models were used to calculate hazard ratios (HRs) with 95% confidence intervals (CIs) for the risk of primary and secondary outcomes in *azithromycin users* compared to *azithromycin non-users*. Sensitivity analyses were performed as described above. All tests were two tailed and *p*-values of ≤ 0.05 were taken to indicate statistical significance. All analyses were performed in the TriNetX platform which incorporates R (v4.3.1, R Foundation for Statistical Foundation, Vienna, Austria).

## Results

The initial cohort of AF patients with COPD was composed by 2442 *azithromycin users* (71.4 ± 10.3 years, 49.1% females) and 58,817 *non-azithromycin users* (73.0 ± 10.3 years, 47.5% females) (Table 1). Before PSM, *azithromycin users* were younger, with a higher prevalence of dyslipidemia and hypertension compared to *azithromycin non-users* (Table 1). After PSM, 2434 patients were successfully matched for each group and no significant differences were found for all the demographic and clinical variables considered (Table 1).

**Table 1:** Baseline characteristics of azithromycin users and non-users before and after propensity score matching

	Before propensity score match			After propensity score match		
	Azithromycin users *n* = 2442	Non-users *n* = 58,817	ASD	Azithromycin users *n* = 2434	Non-users *n* = 2434	ASD
Age, years (± SD)	71.4 ± 10.3	73.0 ± 10.3	0.158	71.4 ± 10.3	71.9 ± 10.5	0.047
Female, *n* (%)	1196 (49.1)	27,713 (47.5)	0.031	1195 (49.1)	1230 (50.5)	0.029
White, *n* (%)	1909 (78.4)	46,729 (80.2)	0.044	1909 (78.4)	1918 (78.4)	0.009
Black or African American, *n* (%)	300 (12.3)	5704 (9.8)	0.081	300 (12.3)	310 (12.7)	0.012
Arterial hypertension, *n* (%)	1968 (80.9)	44,608 (76.5)	0.106	1968 (80.9)	2012 (82.7)	0.047
Diabetes mellitus, *n* (%)	934 (38.4)	21,844 (37.5)	0.018	934 (38.4)	969 (39.8)	0.029
Chronic kidney disease, *n* (%)	659 (27.1)	16,733 (28.7)	0.037	659 (27.1)	733 (30.1)	0.067
Obesity, *n* (%)	677 (27.8)	16,733 (28.7)	0.097	677 (27.8)	715 (29.4)	0.035
Dyslipidemia, *n* (%)	1647 (67.6)	36,151 (62.0)	0.118	1647 (67.6)	1689 (69.4)	0.038
Ischemic heart disease, *n* (%)	1298 (53.3)	30,645 (35.9)	0.018	1298 (53.3)	1397 (57.4)	0.082
Heart failure, *n* (%)	1162 (47.7)	28,915 (49.6)	0.038	1,162 (47.7)	1,254 (51.5)	0.076
Ischemic stroke, *n* (%)	224 (9.2)	5,743 (9.9)	0.022	224 (9.2)	211 (8.7)	0.003
Lipid-lowering drugs, *n* (%)	1628 (66.9)	35,342 (60.6)	0.130	1628 (66.9)	1687 (69.3)	0.053
Beta-blockers, *n* (%)	1706 (70.1)	38,573 (66.2)	0.083	1705 (70.0)	1774 (72.9)	0.063
Diuretics, *n* (%)	1683 (69.1)	38,502 (66.1)	0.065	1683 (69.1)	1760 (72.3)	0.070
Antiarrhythmics, *n* (%)	1428 (58.6)	33,523 (57.5)	0.023	1427 (58.6)	1491 (61.3)	0.054
Calcium channel blockers, *n* (%)	1398 (57.4)	29,489 (50.6)	0.137	1397 (57.4)	1459 (59.9)	0.052
ACE inhibitors, *n* (%)	1100 (45.2)	22,536 (38.7)	0.091	1099 (45.2)	1140 (46.8)	0.034
Angiotensin II inhibitors, *n* (%)	692 (28.4)	14,605 (25.1)	0.076	692 (28.4)	739 (30.4)	0.043
Anticoagulants, *n* (%)	1844 (75.7)	41,698 (71.5)	0.095	1843 (75.7)	1912 (78.6)	0.009
Antiplatelets, *n* (%)	1602 (65.8)	34,271 (58.8)	0.145	1601 (65.8)	1678 (68.9)	0.068

*ASD* Absolute standardized mean difference

### Risk of all cardiovascular events after exacerbation of COPD

**Fig. 1:**
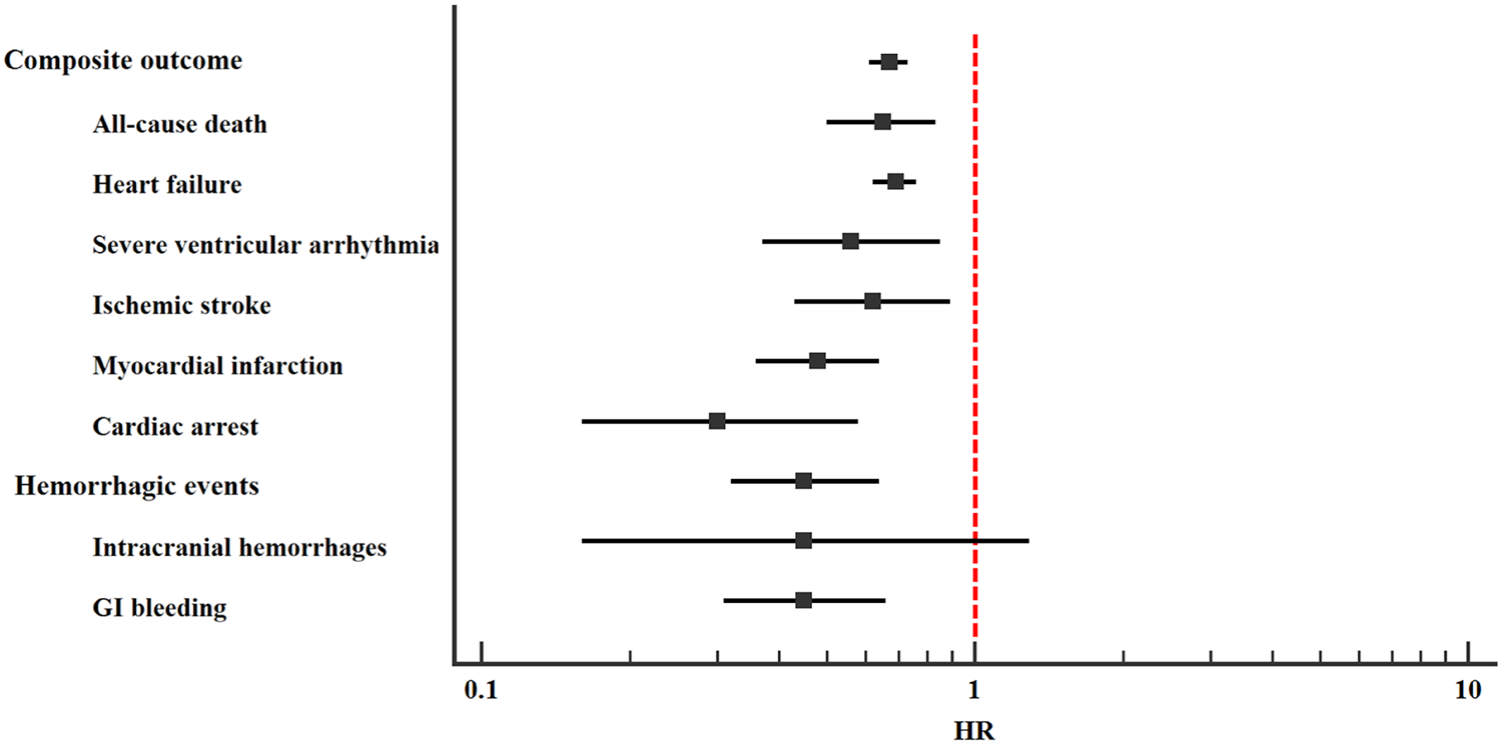
Risk of composite outcome and hemorrhagic events in *azithromycin users* compared to *azithromycin non-users*

After PSM, the total number of composite cardiovascular and hemorrhagic outcomes reported 30 days after the last COPD acute exacerbation was 761 (31.1%) and 46 (1.9) in *azithromycin users,* and 1026 (42.2%) and 99 (3.6%) in *azithromycin non-users* (HR 0.67, 95% CI 0.61–0.73 and HR 0.45, 95% CI 0.32–0.64, respectively. Table 2, Fig. 1). Analyzing the secondary outcomes, *azithromycin users* showed a lower risk of all-cause death (HR 0.65, 95% CI 0.50–0.83), heart failure (HR 0.69, 95% CI 0.62–0.76), ventricular arrhythmias (HR 0.56, 95% CI 0.37–0.85), ischemic stroke (HR 0.62, 95% CI 0.43–0.89), myocardial infarction (HR 0.48, 95% CI 0.36–0.64), cardiac arrest (HR 0.30, 95% CI 0.16–0.58), and GI bleeding (HR 0.45, 95% CI 0.32–0.66) compared to *azithromycin non-users* (Table 2, Fig. 1). No significant difference for the 30 days risk of ICH was found between the two groups (HR 0.45, 95%CI 0.16–1.29) (Table 2, Fig. 1).

**Table 2:** 30-day risk of cardiovascular events after COPD exacerbation in azithromycin users and non-users

	Azithromycin users (*n* = 2434)	Non-users (*n* = 2434)	
	Events, *n* (%)	Events, n (%)	HR (95%CI)
Composite outcome	761 (31.1)	1,026 (42.2)	0.67 (0.61–0.73)
All-cause death	100 (4.1)	152 (6.2)	0.65 (0.50–0.83)
Heart failure	635 (26.1)	846 (34.8)	0.69 (0.62–0.76)
Severe ventricular arrhythmias	34 (1.4)	60 (2.5)	0.56 (0.37–0.85)
Ischemic Stroke	46 (1.9)	73 (3.0)	0.62 (0.43–0.89)
Myocardial infarction	67 (2.8)	137 (5.6)	0.48 (0.36–0.64)
Cardiac arrest	12 (0.5)	39 (1.6)	0.30 (0.16–0.58)
Hemorrhagic events	46 (1.9)	99 (4.1)	0.45 (0.32–0.64)
Intracranial hemorrhage	10 (0.4)	11 (0.5)	0.45 (0.16–1.29)
GI bleeding	41 (1.7)	88 (3.6)	0.45 (0.31–0.66)

*HR* Hazard Ratio, *CI* Confidence Interval, *GI* Gastrointestinal

### Risk of cardiovascular events during the different time frames.

In time-stratified analyses, the beneficial effect of azithromycin in reducing the risk of cardiovascular outcome was highest during the 1st week (HR 0.57, 95% CI 0.50–0.65), and progressively decreases during the 2nd week (HR 0.69, 95% CI 0.59–0.80) and the 3rd–4th weeks (HR 0.76, 95% CI 0.68–0.88) (Table 3). The same trend could be observed for the risks of all the other secondary outcomes, where greatest effect was observed during the first 7 days after exacerbation (Table 3).

**Table 3:** Risk of early cardiovascular complications after COPD exacerbation in azithromycin users and non-users during the different time frames

		1st week			2nd week			3rd–4th weeks	
	Azithromycin Users	Azithromycin non-users		Azithromycin Users	Azithromycin non-users		Azithromycin Users	Azithromycin non-users	
	Events *n* (%)	Events *n* (%)	HR 95%CI	Events *n* (%)	Events *n* (%)	HR 95%CI	Events *n* (%)	Events *n* (%)	HR 95%CI
Composite outcome	353 (14.5)	587 (24.1)	0.57 (0.50–0.65)	282 (11.6)	393 (16.1)	0.69 (0.59–0.80)	453 (18.6)	552 (22.7)	0.76 (0.68–0.88)
All-cause death	30 (1.2)	50 (2.1)	0.60 (0.38–0.94)	29 (1.2)	42 (1.7)	0.68 (0.42–1.09)	41 (1.7)	60 (2.5)	0.66 (0.45–0.99)
Heart failure	302 (12.4)	497 (20.4)	0.58 (0.50–0.67)	240 (9.9)	319 (13.1)	0.78 (0.62–0.86)	381 (15.7)	451 (18.5)	0.81 (0.70–0.92)
Severe ventricular arrhythmias	13 (0.5)	29 (1.2)	0.45 (0.23–0.86)	10 (0.4)	21 (0.9)	0.42 (0.19–0.92)	460 (3.4)	489 (3.6)	0.65 (0.34–1.22)
Ischemic Stroke	21 (0.9)	41 (1.7)	0.51 (0.30–0.86)	14 (0.6)	19 (0.8)	0.73 (0.36–1.45)	20 (0.8)	30 (1.6)	0.50 (0.29–0.85)
Myocardial infarction	31 (1.3)	71 (2.9)	0.43 (0.28–0.66)	19 (0.8)	45 (1.8)	0.41 (0.24–0.71)	34 (1.4)	51 (2.1)	0.65 (0.42–1.00)
Cardiac arrest	10 (0.4)	21 (0.9)	0.19 (0.07–0.55)	10 (0.4)	13 (0.5)	0.38 (0.14–1.07)	10 (0.4)	13 (0.5)	0.53 (0.21–1.32)
Hemorrhagic events	12 (0.5)	51 (2.1)	0.23 (0.12–0.44)	16 (0.7)	41 (1.7)	0.38 (0.22–0.68)	27 (1.1)	41 (1.7)	0.64 (0.39–1.04)
ICH*	10 (0.4)	10 (0.4)	0.14 (0.02–1.16)	–	–	–	–	–	–
GI bleeding	11 (0.5)	44 (1.8)	0.25 (0.13–0.48)	15 (0.6)	36 (1.5)	0.41 (0.22–0.75)	23 (0.9)	36 (1.5)	0.62 (0.37–1.05)

*HR* Hazard Ratio, *CI* Confidence Interval, *ICH* intracranial hemorrhage, *GI* Gastrointestinal

^*^No HRs were calculated for ICH in the second and third period because only 1 event occurred

### Sensitivity analyses

**Fig. 2:**
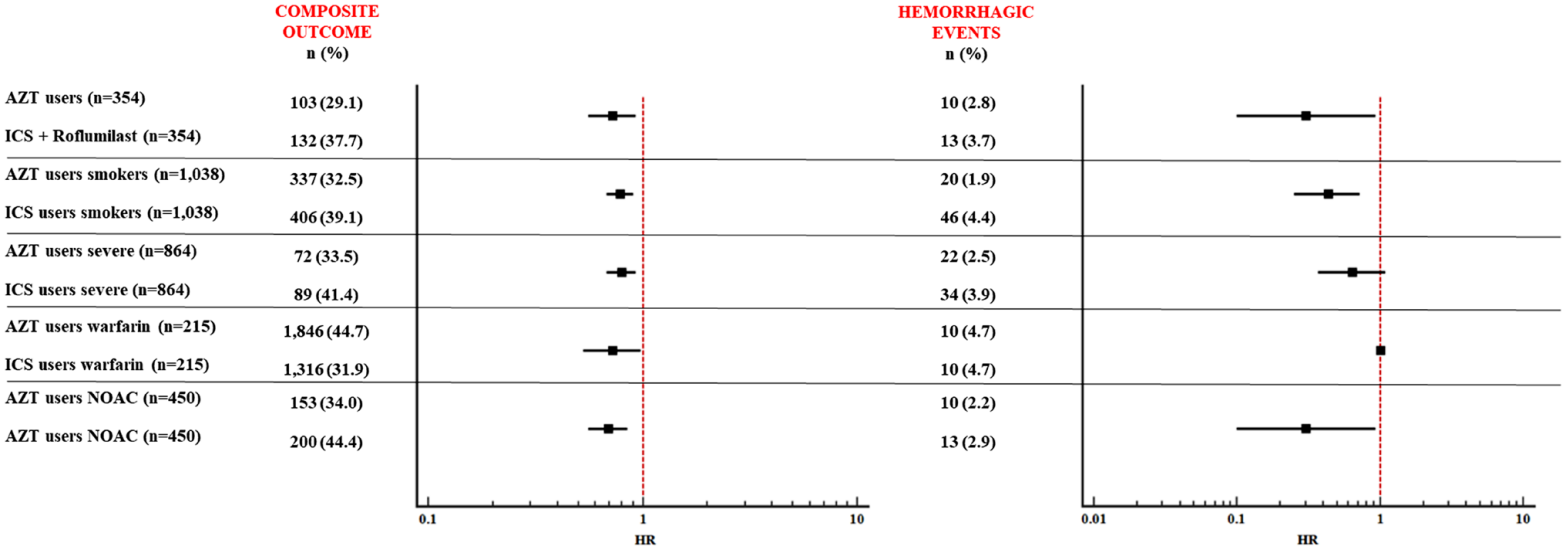
Sensitivity analyses after propensity score match

In the analysis performed to assess the 30-day risk of adverse events in COPD azithromycin users compared to non-users receiving inhaled corticosteroids and roflumilast, after PSM, 103 (29.1%) *azithromycin users* and 132 (37.7%) *roflumilast users* experienced the cardiovascular outcome within 30 days after COPD exacerbation (HR 0.72, 95% CI 0.56–0.93), whereas 10 (2.8%) and 13 (3.7%) experienced the hemorrhagic outcomes, respectively (HR 0.30, 95% CI 0.10–0.93) (Fig. 2).

In analyses restricted to smokers, PSM successfully matched 1038 well-balanced patients to each group. 337 (32.5%) *azithromycin users* and 406 (39.1%) *azithromycin non-users* experienced a cardiovascular composite event within 30 days after COPD exacerbation (HR 0.43, 95% CI 0.25–0.72), whereas 20 (1.9%) and 46 (4.4%) experienced the hemorrhagic outcome (HR 0.43, 95% CI 0.25–0.72). Thus, suggesting that the primary analysis was robust regardless of smoking status (Fig. 2).

In analyses restricted to severe exacerbations, PSM successfully matched 864 to each group. The composite event occurred within 30 days of index event in 72 (33.5%) *azithromycin users* and 89(41.4%) *azithromycin non-users* (HR 0.72, 95% CI 0.53–0.98), whereas 22 (2.5%) and 34 (3.9%) experienced a hemorrhagic event, respectively (HR 0.64, 95% CI 0.37–1.09). Again, suggesting the primary analysis was robust (Fig. 2).

In the analyses performed considering separately warfarin and NOAC users we found that the 30-day risk of composite outcome in azithromycin users was reduced, and no increased risk of hemorrhagic events was found, for both the treatments (Fig. 2). In *azithromycin users* treated with warfarin, the HR of composite cardiovascular outcome was 0.72 (95% CI 0.53–0.98) when compared to *azithromycin non-users*, whereas no difference in the number of hemorrhagic events (10 vs 10) was recorded (Fig. 2). Compared to *azithromycin non-users, azithromycin users* treated with NOAC the HR of composite cardiovascular outcome was 0.69 (95% CI 0.56–0.85), whereas that for hemorrhagic events was 0.30 (95% CI 0.10–0.93) (Fig. 2).

## Discussion

In this study, the principal findings are as follows: (1) After COPD exacerbations, AF patients treated with ICS and low-dose azithromycin prophylaxis have a reduced 30-day risk of all-cause death, cardiovascular events and bleeding compared to those treated only with ICS; (2) The highest risk reduction for both the composite cardiovascular and hemorrhagic events was observed during the first week after the COPD exacerbation; and (3)The beneficial effect of low-dose azithromycin prophylaxis was robust across a number of sensitivity analyses.

Long-term macrolide therapy is recommended by multiple guidelines for COPD patients suffering frequent exacerbation with high quality evidence from multiple randomized clinical trials and meta-analyses demonstrating reduced exacerbation rates associated with treatment [14–16]. However, there is conflicting evidence as to the cardiovascular safety of macrolide therapy. For example, the Federal Drug Administration placed a risk warning for ventricular arrythmias based on an observational study which suggested increased risk of cardiovascular death, but not all-cause death, 10 days after initiation of a course of azithromycin for acute indications when compared to amoxicillin [9]. A population based retrospective cohort study of people with non-tuberculous mycobacteria pulmonary disease in South Korea observed increased cardiovascular disease incidence in macrolide-treated patients [17]. However, no increased risk was observed in a large Danish national cohort study on COPD patients [18] and no adverse cardiovascular disease signal was reported in the RCT of macrolides in COPD or bronchiectasis [15, 19].

COPD represents one of the pivotal factors in determining the risk of death and cardiovascular events in AF patients already treated with OAC [1]. The relationship between AF and COPD is bidirectional, the AF onset in COPD patients is associated with a worse quality of life and an increased risk of respiratory failure [20], whereas the COPD exacerbations in AF patients dramatically increase the risk of cardiovascular events [21]. This increase is the results of different mechanisms, (i) hypoxia and hypercapnia induce vasoconstriction in the pulmonary circulation with pulmonary hypertension, (ii) the inflammatory response can precipitate thrombotic events, and (iii) the hyper sympathetic stimulation necessary to relax the airways’ smooth muscle cells triggers life-threatening arrhythmias and induce ischemic events [22].

In this context, the anti-inflammatory mechanisms of action by which macrolides are thought to reduce exacerbation risk that include improved efferocytosis and suppression of inflammatory cytokines such as IL-6, IL-1 and reduced TNF-α, may provide a suggestive explanation of the protective effect we found in *azithromycin users*. Indeed, the reduced inflammatory state associated with low-dose azithromycin prophylaxis in AF patients with COPD, could lead to less severe exacerbations and thus to a reduced risk of cardiovascular events and death. Hence, this suggests that azithromycin’s anti-inflammatory effect may be additive to that of ICS. Moreover, the reduced risk of adverse events was consistent also in the sensitivity analyses: (i) using as comparator COPD patients treated with ICS and roflumilast, another anti-inflammatory drug that exerts its effect through a different mechanism than azithromycin (i.e., inhibiting phosphodiesterase), (ii) in patients with an additional source of oxidative stress, inflammation, and sympathetic agonism such as the smokers, in which some guidelines do not recommend use of macrolides [7]; and (iii) only in those with severe COPD exacerbations, that were at higher risk per se. These subgroup analyses demonstrate the robustness of our findings.

Beyond the cardiovascular safety profile, another source of possible concerns for macrolides use in AF patients with COPD is the possible presence of drug interactions. Indeed, macrolides are a well-known class of CYP3A4 and p-glycoprotein inhibitors and may perturbate the plasma concentration of both vitamin-K antagonists and NOACs [23]. In our study, we showed that not only the risk of cardiovascular events, but also the hemorrhagic risk was reduced in *azithromycin users,* and this was irrespective of the type of oral anticoagulant (warfarin or NOACs). A partial explanation behind the reduced risk of bleeding in COPD patients with AF *azithromycin users* could lie in the reduced incidence of collateral complications triggered by COPD exacerbation that could increase the hemorrhagic risk. COPD exacerbations are often associated with hypertensive crisis that could facilitate vascular rupture and hemorrhagic events, the respiratory distress facilitates the onset of dehydration that in turn can facilitate the onset of acute renal failure and reduced OAC clearance, moreover, both the hypoxemia and the hypercapnia can cause delirium in the elderly with subsequent risk of falls and traumatisms.

This body of evidence underlines the complexity of AF patients with COPD and the importance of reducing the background inflammatory state associated with COPD to counteract the risk adverse events. This could be reached through the optimal management not only of COPD but also of all the comorbidities that often coexist in AF patients.

The latest management guidelines for AF management [24, 25] advocate this type of holistic approach and proposing the atrial fibrillation better care (ABC) pathway as a possible tool drives the clinical decisions [26]. The ABC pathway is based on the 3 main pillars: “A” avoid stroke with oral anticoagulation, “B” better management of the symptoms with a patient-centered symptom-directed decisions on rate or rhythm control; and “C” Cardiovascular risk factor optimization and lifestyle changes [25]. Adherence to the ABC pathway was associated with a reduced risk of all-cause death, cardiovascular events, and AF-related hospitalization in different populations [27, 28], even in complex phenotypes [29]. One of the main concepts sustained by this integrated approach is that the risk of adverse events in already anticoagulated AF patients is due to all the cardiovascular comorbidities that often coexist in AF patients [26]. Although a schematic approach has been proposed to encode the optimal management of common cardiovascular comorbidities [28], no specific treatment associated with a reduced risk of cardiovascular events was found for COPD. In this context, the possible use of low-dose azithromycin prophylaxis in AF patients with COPD could represent the possible missing piece of this puzzle. Indeed, reducing the rate and the severity of COPD exacerbation seems to be associated with an overall risk reduction for all the related adverse events.

### Limitations

This study was observational and retrospective, and despite successful matching, we cannot rule out residual bias or indication bias related to unmeasured confounders. Outcomes in this study were based on ICD-10 codes and as such are unadjudicated and may be prone to misclassification or under-reporting. Similarly, azithromycin prescriptions were identified using RxNorm codes which may be prone to the same limitations. We did not adjust the analyses for procedures or treatments administered after the index event to avoid the possibility of introducing immortal time bias. Overall, results here are hypothesis generating and robust prospective studies are needed to validate our findings.

## Conclusion

Our data suggest that long-term azithromycin prophylaxis is associated with reduced 30-day risk of cardiovascular events associated with exacerbations in AF patients with COPD. Further studies are needed to prospectively validate these findings, understand mechanisms, and delineate those most likely to benefit.

## Supplementary Information

Below is the link to the electronic supplementary material.

Supplementary file1 (DOCX 19 KB)

## Data Availability

The data that support the findings of this study are available from the corresponding author upon reasonable request.

## References

[1] Romiti GF, Corica B, Pipitone E, Vitolo M, Raparelli V, Basili S, Boriani G, Harari S, Lip GYH, Proietti M, Group A-CIC (2021) Prevalence, management and impact of chronic obstructive pulmonary disease in atrial fibrillation: a systematic review and meta-analysis of 4,200,000 patients. Eur Heart J 42(35):3541–3554 34333599 10.1093/eurheartj/ehab453

[2] Raparelli V, Pastori D, Pignataro SF, Vestri AR, Pignatelli P, Cangemi R, Proietti M, Davi G, Hiatt WR, Lip GYH, Corazza GR, Perticone F, Violi F, Basili S, Collaborators AS (2018) Major adverse cardiovascular events in non-valvular atrial fibrillation with chronic obstructive pulmonary disease: the ARAPACIS study. Intern Emerg Med 13(5):651–660 29582316 10.1007/s11739-018-1835-9

[3] Proietti M, Laroche C, Drozd M, Vijgen J, Cozma DC, Drozdz J, Maggioni AP, Boriani G, Lip GY, Investigators E-A (2016) Impact of chronic obstructive pulmonary disease on prognosis in atrial fibrillation: a report from the EURObservational research programme pilot survey on atrial fibrillation (EORP-AF) general registry. Am Heart J 181:83–91 27823697 10.1016/j.ahj.2016.08.011

[4] Bucci T, Romiti GF, Shantsila A, Teo WS, Park HW, Shimizu W, Corica B, Proietti M, Tse HF, Chao TF, Frost F, Lip GYH (2024) Asia-Pacific heart rhythm society atrial fibrillation registry I. Risk of death and cardiovascular events in Asian patients with atrial fibrillation and chronic obstructive pulmonary disease: a report from the prospective APHRS registry. J Am Heart Assoc 13(7):e032785 38533983 10.1161/JAHA.123.032785 PMC11179754

[5] Dransfield MT, Criner GJ, Halpin DMG, Han MK, Hartley B, Kalhan R, Lange P, Lipson DA, Martinez FJ, Midwinter D, Singh D, Wise R, Kunisaki KM (2022) Time-dependent risk of cardiovascular events following an exacerbation in patients with chronic obstructive pulmonary disease: post hoc analysis from the IMPACT trial. J Am Heart Assoc 11(18):e024350 36102236 10.1161/JAHA.121.024350 PMC9683674

[6] Rothnie KJ, Connell O, Mullerova H, Smeeth L, Pearce N, Douglas I, Quint JK (2018) Myocardial infarction and ischemic stroke after exacerbations of chronic obstructive pulmonary disease. Ann Am Thorac Soc 15(8):935–946 29723057 10.1513/AnnalsATS.201710-815OC PMC6322039

[7] Agusti A, Celli BR, Criner GJ, Halpin D, Anzueto A, Barnes P, Bourbeau J, Han MK, Martinez FJ, de Oca MM, Mortimer K, Papi A, Pavord I, Roche N, Salvi S, Sin DD, Singh D, Stockley R, Varela MVL, Wedzicha JA, Vogelmeier CF (2023) Global initiative for chronic obstructive lung disease 2023 report: GOLD executive summary. Respirology 28(4):316–338 36856440 10.1111/resp.14486

[8] (NICE) NIfHaCE (2019) Chronic obstructive pulmonary disease in over 16s: diagnosis and management. Chronic obstructive pulmonary disease in over 16s: diagnosis and management, London, 2019.

[9] Ray WA, Murray KT, Hall K, Arbogast PG, Stein CM (2012) Azithromycin and the risk of cardiovascular death. N Engl J Med 366(20):1881–1890 22591294 10.1056/NEJMoa1003833 PMC3374857

[10] Ridker PM, Everett BM, Thuren T, MacFadyen JG, Chang WH, Ballantyne C, Fonseca F, Nicolau J, Koenig W, Anker SD, Kastelein JJP, Cornel JH, Pais P, Pella D, Genest J, Cifkova R, Lorenzatti A, Forster T, Kobalava Z, Vida-Simiti L, Flather M, Shimokawa H, Ogawa H, Dellborg M, Rossi PRF, Troquay RPT, Libby P, Glynn RJ, Group CT (2017) Antiinflammatory therapy with canakinumab for atherosclerotic disease. N Engl J Med 377(12):1119–1131 28845751 10.1056/NEJMoa1707914

[11] Bucci T, Wat D, Nazareth D, Sibley S, Wootton D, Lip GY, Frost F (2024) Risk of cardiovascular events after acute exacerbations of chronic obstructive pulmonary disease in patients receiving long-term low-dose azithromycin. Am J Respir Crit Care Med. 10.1164/rccm.202309-1699LE 38502239 10.1164/rccm.202309-1699LE

[12] Patel AR, Kowlessar BS, Donaldson GC, Mackay AJ, Singh R, George SN, Garcha DS, Wedzicha JA, Hurst JR (2013) Cardiovascular risk, myocardial injury, and exacerbations of chronic obstructive pulmonary disease. Am J Respir Crit Care Med 188(9):1091–1099 24033321 10.1164/rccm.201306-1170OC PMC3863745

[13] Han MK, Tayob N, Murray S, Dransfield MT, Washko G, Scanlon PD, Criner GJ, Casaburi R, Connett J, Lazarus SC, Albert R, Woodruff P, Martinez FJ (2014) Predictors of chronic obstructive pulmonary disease exacerbation reduction in response to daily azithromycin therapy. Am J Respir Crit Care Med 189(12):1503–1508 24779680 10.1164/rccm.201402-0207OC PMC4226018

[14] Naderi N, Assayag D, Mostafavi-Pour-Manshadi SM, Kaddaha Z, Joubert A, Ouellet I, Drouin I, Li PZ, Bourbeau J (2018) Long-term azithromycin therapy to reduce acute exacerbations in patients with severe chronic obstructive pulmonary disease. Respir Med 138:129–136 29724384 10.1016/j.rmed.2018.03.035

[15] Albert RK, Connett J, Bailey WC, Casaburi R, Cooper JA Jr, Criner GJ, Curtis JL, Dransfield MT, Han MK, Lazarus SC, Make B, Marchetti N, Martinez FJ, Madinger NE, McEvoy C, Niewoehner DE, Porsasz J, Price CS, Reilly J, Scanlon PD, Sciurba FC, Scharf SM, Washko GR, Woodruff PG, Anthonisen NR, Network CCR (2011) Azithromycin for prevention of exacerbations of COPD. N Engl J Med 365(8):689–698 21864166 10.1056/NEJMoa1104623 PMC3220999

[16] Herath SC, Normansell R, Maisey S, Poole P (2018) Prophylactic antibiotic therapy for chronic obstructivea pulmonary disease (COPD). Cochrane Database Syst Rev 10(10):CD009764 30376188 10.1002/14651858.CD009764.pub3 PMC6517028

[17] Kang J, Kim YJ, Shim TS, Jo KW (2018) Risk for cardiovascular disease in patients with nontuberculous mycobacteria treated with macrolide. J Thorac Dis 10(10):5784–5795 30505486 10.21037/jtd.2018.09.145 PMC6236155

[18] Svanstrom H, Pasternak B, Hviid A (2013) Use of azithromycin and death from cardiovascular causes. N Engl J Med 368(18):1704–1712 23635050 10.1056/NEJMoa1300799

[19] Serisier DJ, Martin ML, McGuckin MA, Lourie R, Chen AC, Brain B, Biga S, Schlebusch S, Dash P, Bowler SD (2013) Effect of long-term, low-dose erythromycin on pulmonary exacerbations among patients with non-cystic fibrosis bronchiectasis: the BLESS randomized controlled trial. JAMA 309(12):1260–1267 23532242 10.1001/jama.2013.2290

[20] Chen CY, Liao KM (2018) The impact of atrial fibrillation in patients with COPD during hospitalization. Int J Chron Obstruct Pulmon Dis 13:2105–2112 30022816 10.2147/COPD.S166534 PMC6044355

[21] Wang M, Lin EP, Huang LC, Li CY, Shyr Y, Lai CH (2020) Mortality of cardiovascular events in patients with COPD and preceding hospitalization for acute exacerbation. Chest 158(3):973–985 32184108 10.1016/j.chest.2020.02.046

[22] Simons SO, Elliott A, Sastry M, Hendriks JM, Arzt M, Rienstra M, Kalman JM, Heidbuchel H, Nattel S, Wesseling G, Schotten U, van Gelder IC, Franssen FME, Sanders P, Crijns H, Linz D (2021) Chronic obstructive pulmonary disease and atrial fibrillation: an interdisciplinary perspective. Eur Heart J 42(5):532–540 33206945 10.1093/eurheartj/ehaa822

[23] Yagi T, Mannheimer B, Reutfors J, Ursing J, Giunta DH, Kieler H, Linder M (2023) Bleeding events among patients concomitantly treated with direct oral anticoagulants and macrolide or fluoroquinolone antibiotics. Br J Clin Pharmacol 89(2):887–897 36098510 10.1111/bcp.15531 PMC10092847

[24] Chao TF, Joung B, Takahashi Y, Lim TW, Choi EK, Chan YH, Guo Y, Sriratanasathavorn C, Oh S, Okumura K, Lip GYH (2022) 2021 focused update consensus guidelines of the Asia pacific heart rhythm society on stroke prevention in atrial fibrillation: executive summary. Thromb Haemost 122(1):20–47 34773920 10.1055/s-0041-1739411 PMC8763451

[25] Hindricks G, Potpara T, Dagres N, Arbelo E, Bax JJ, Blomstrom-Lundqvist C, Boriani G, Castella M, Dan GA, Dilaveris PE, Fauchier L, Filippatos G, Kalman JM, La Meir M, Lane DA, Lebeau JP, Lettino M, Lip GYH, Pinto FJ, Thomas GN, Valgimigli M, Van Gelder IC, Van Putte BP, Watkins CL, Group ESCSD (2021) 2020 ESC Guidelines for the diagnosis and management of atrial fibrillation developed in collaboration with the European association for cardio-thoracic surgery (EACTS): the task force for the diagnosis and management of atrial fibrillation of the European society of cardiology (ESC) developed with the special contribution of the European heart rhythm association (EHRA) of the ESC. Eur Heart J 42(5):373–498 32860505 10.1093/eurheartj/ehaa612

[26] Lip GYH (2017) The ABC pathway: an integrated approach to improve AF management. Nat Rev Cardiol 14(11):627–628 28960189 10.1038/nrcardio.2017.153

[27] Romiti GF, Pastori D, Rivera-Caravaca JM, Ding WY, Gue YX, Menichelli D, Gumprecht J, Koziel M, Yang PS, Guo Y, Lip GYH, Proietti M (2022) Adherence to the “atrial fibrillation better care” pathway in patients with atrial fibrillation: impact on clinical outcomes-a systematic review and meta-analysis of 285,000 patients. Thromb Haemost 122(3):406–414 34020488 10.1055/a-1515-9630

[28] Bucci T, Proietti M, Shantsila A, Romiti GF, Teo WS, Park HW, Shimizu W, Tse HF, Lip GYH, Chao TF, Investigators A-AR (2023) Integrated care for atrial fibrillation using the ABC pathway in the prospective APHRS-AF registry. JACC Asia 3(4):580–591 37614548 10.1016/j.jacasi.2023.04.008 PMC10442886

[29] Krittayaphong R, Treewaree S, Wongtheptien W, Kaewkumdee P, Lip GYH (2023) Clinical phenotype classification to predict risk and optimize the management of patients with atrial fibrillation using the atrial fibrillation better care (ABC) pathway: a report from the COOL-AF registry. QJM. 10.1093/qjmed/hcad219 10.1093/qjmed/hcad219 37788118

