# Supplementary material for: Low-dose azithromycin prophylaxis in patients with atrial fibrillation and chronic obstructive pulmonary disease
Source: Intern Emerg Med. 2024 May 31;19(6):1615–23. doi: 10.1007/s11739-024-03653-0 (PMC11405424; doi:10.1007/s11739-024-03653-0)
Supplement: Supplementary file 1 — Supplementary file1 (DOCX 19 KB) [file 11739_2024_3653_MOESM1_ESM.docx]

**Risk of death, cardiovascular events and bleeding in patients with atrial fibrillation and chronic obstructive pulmonary disease treated with** **low-dose azithromycin prophylaxis.**

Tommaso Bucci, Dennis Wat, Sarah Sibley, Dan Wootton, David Green, Pasquale Pignatelli, Gregory Y. H. Lip, Freddy Frost

**Supplementary Material**

**Supplementary detail regarding methods**

TriNetx is a research network utilized for several scientific purposes, compliant with the Health Insurance Portability and Accountability Act and the US federal law which protects the privacy and security of healthcare data, including de-identified data as per the de-identification standard of the HIPAA Privacy Rule(https://trinetx.com/real-world-resources/publications/). To gain access to the data in the TriNetX research network, requests are directed to TriNetX and a data sharing agreement is required. As a federated research network, studies using the TriNetX health research network do not need ethical approval as no patient identifiable identification is received. The data are stored on the TriNetX database via a physical server at the institution’s data centre or a virtual hosted appliance. The TriNetX platform comprises of a series of these appliances connected into a federated network. This network can broadcast queries to each appliance. Results are subsequently collected and aggregated. Once the data are sent to the network, it is mapped to a standard and controlled set of clinical terminologies and undergoes a data quality assessment including ‘data cleaning’ that rejects records which do not meet the TriNetX quality standards. The TriNetX database performs internal and extensive data quality assessment with every refresh based on conformance, completeness, and plausibility (http://doi.org/10.13063/2327-9214.1244). HIPAA (Health Insurance Portability and Accountability Act) compliance of the clinical patient data is achieved using deidentification. Available data types within the network include demographics, diagnoses (represented by ICD-10-CM codes), procedures (coded in ICD-10-PCS or CPT), and measurements (coded to LOINC). While extensive information is provided about patients’ diagnoses and procedures, other variables (such as socioeconomic and lifetime factors are not comprehensively represented). The advantage of electronic health record data over insurance claim data is that both insured and uninsured patients are included. An advantage of electronic health record data over survey data is that the former represents the diagnostic rates in the population presenting to healthcare facilities. This provides an accurate account of the burden of specific diagnoses on healthcare systems. One primary limitation of relying on diagnoses is that they do not account for undiagnosed patients who might have a condition but have not yet received medical support. Another general limitation of electronic health record data is that a patient may be seen in different healthcare organizations for different components of their care. If one healthcare organization is not part of the federated network, then part of their medical records may not be available. Using a network of healthcare organizations, rather than a single site, limits this possibility but does not fully remove it. Propensity Score Matched Analyses Using logistic regression [Logistic Regression of the scikit-learn package in Python (version 3.7)], TriNetX performs a 1:1 greedy nearest neighbor matching model, with a caliper of 0.1 pooled standard deviations. To eliminate bias resulting from nearest neighbour algorithms, the orders of rows are randomized. Any baseline characteristic with a standardised mean difference between cohorts lower than 0.1 is deemed well matched (https://www.tandfonline.com/doi/full/10.1080/00273171.2011.568786).

Supplementary Table 1. ICD-10-CM codes for identify cardiovascular events after acute exacerbations.

| **Primary outcomes** | **Early cardiovascular events** | **ICD-10-CM-codes** |
| --- | --- | --- |
| **Composite outcome** | All-cause death | - Deceased (variable codified by TriNetX). |
|  | Heart Failure | - I50 Heart failure |
|  | Severe ventricular arrhythmia | The composite of any of the following:   - I47.2 Ventricular tachycardia. - I49.0 Ventricular fibrillation or flutter. |
|  | Ischemic stroke or transient cerebral ischemic attack | The composite of any of the following:   - I63 Cerebral Infarction. - G45 Transient cerebral ischemic attacks and related syndromes. |
|  | Myocardial infarction | - I21 Acute myocardial infarction - I20.0 Unstable angina |
| **Hemorrhagic events** | Intracranial haemmorhage | - I60 Nontraumatic subarachnoid hemorrhage - I61 Nontraumatic intracerebral hemorrhage - I63 Other and unspecified nontraumatic intracranial hemorrhage |
|  | Gastrointestinal bleeding | - K92.1 Melena - K92.0 Hematemesis - K92.2 Gastrointestinal hemorrhage, unspecified |
